# Technical aspects of fourth ventricle ependymomas in adults: how I do it

**DOI:** 10.1007/s00701-024-06121-y

**Published:** 2024-05-21

**Authors:** Adéla Bubeníková, Vladimír Beneš

**Affiliations:** 1 Department of Neurosurgery and Neurooncology, 1St Faculty of Medicine, Charles University and Military University Hospital, U Vojenské Nemocnice 1200, 169 02 Prague 6, Czech Republic; 2 Department of Neurosurgery, 2Nd Faculty of Medicine, Charles University and Motol University Hospital, U Vojenské Nemocnice 1200, 169 02 Prague 6, Czech Republic

**Keywords:** Ependymoma, Surgical resection, Microsurgery, Technical note, Case series

## Abstract

**Background:**

Ependymomas in the fourth ventricle in adults are rare entity. Surgical treatment of adult ependymomas is the only treatment modality since no other effective alternative is available. Radical resection often means cure but it is hindered by the nature and location of the lesion.

**Methods:**

Technical aspects of the fourth ventricle ependymoma surgery in adults are discussed. Anatomy of the area is provided with the step-by-step surgical algorithm.

**Conclusion:**

Radical resection of low-grade ependymoma with a detailed understanding of the anatomy in this area is vital considering the high effectiveness of the treatment and its excellent prognosis.

## Relevant surgical anatomy

Ependymomas of the fourth ventricle in adults are rare tumors composing 2–3% of all intracranial tumors in adults [6, 7]. The vast majority are ependymomas gr. II. These tumors are radioresistant with no available chemotherapy, thus remain purely surgical entity [3]. It also is well established that radical resection is the definitive treatment. The recurrences are rare, most of them being not a true recurrence but rather a growth of the tumor remnant.

**Fig. 1:**
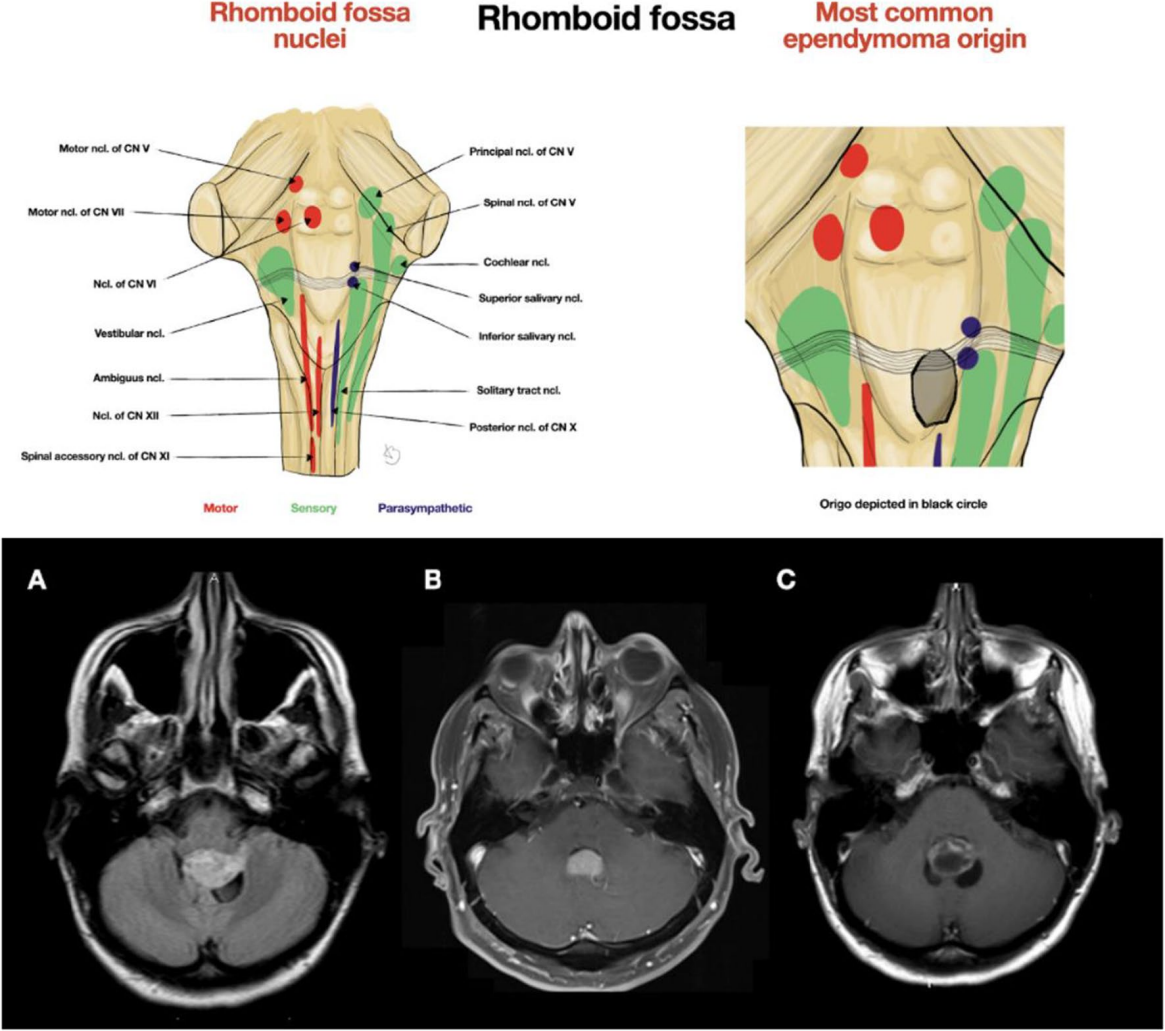
Depiction of the rhomboid fossa nuclei with the most common ependymoma origin from the rhomboid fossa demonstrated with usual close relation to the salivary nuclei. Demonstration of the craniotomy adjustment to the tumor characteristics is provided below. Both (**B**) and (**C**) are ependymomas restricted to the midline, while ependymoma in (**A**) has more extensive behavior to the left side protruding into the Luschka foramen, therefore, craniotomy was extended to this side

The IVth ventricle is the most inferiorly located cerebral ventricle, directing cerebrospinal fluid (CSF) into the central canal of the spinal cord. Superiorly, the aqueduct of Sylvius connects the third with the fourth ventricle. The fourth ventricle is bordered by the rhomboid fossa anteriorly (Fig. 1), by the fastigium, inferior and superior medullary vellum posteriorly, and by the obex inferiorly. Laterally are located cerebellar peduncles. Circulation of the CSF is not directed only to the spinal canal, but also through the foramina of Luschka to the cerebellopontine (CP) cistern, and the foramen of Magendie to the cisterna magna. Choroid plexus along the tela choroidea may protrude out of the lateral foramina of Luschka, forming Bochdalek's flower basket [4, 5].

The most clinically significant vascular structures needed to be held in mind are posterior inferior cerebellar artery (PICA), vertebral artery (VA), anterior inferior cerebellar artery (AICA) in the cerebellopontine angle (CPA), venous sinuses (occipital, marginal, sigmoid) and cerebellar perforating veins.

The first fourth ventricle lesions were resected via transvermian approach [1], while telovelar approach was utilized more recently, avoiding damage to surrounding structures since approaching the ventricle from the inferior, rather than superior [2]. This article deals with technical aspects of the surgical treatment of fourth ventricle ependymomas in adults, providing useful step-by-step navigation with discussing its pros and cons.

## Demographic data

Retrospective analysis of 31 IVth ventricles ependymomas treated at our institution between 1999–2023 was performed. The mean age of included patients was 50.2 ± 17.2 years. Only two ependymomas were CNS WHO grade 3 [6], while the vast majority was CNS WHO grade 2.

## Indications

The vast majority of patients require surgical intervention due to intracranial hypertension, developing hydrocephalus, cerebellar symptoms or neurological deficit.

## Preoperative considerations

### Positioning and skin incision

The patient is intubated, and monitoring devices are introduced, using motor (MEP), somatosensory (SSEP), brainstem (BAEP) evoked potentials and cranial nerve monitoring (V–XII) by a neurophysiologist. The patient is settled in a prone position, Mayfield clamp is fixed, and the head is flexed forward. The goal is to expose the posterior atlantic tubercle as the highest point. Skin incision runs in the midline from the external occipital protuberance down to the spinous process of the C2. Dorsal cervical muscles are split in the midline and reflected laterally, the posterior arch of C1, eventually even C2 are exposed.

### Craniotomy

**Fig. 2:**
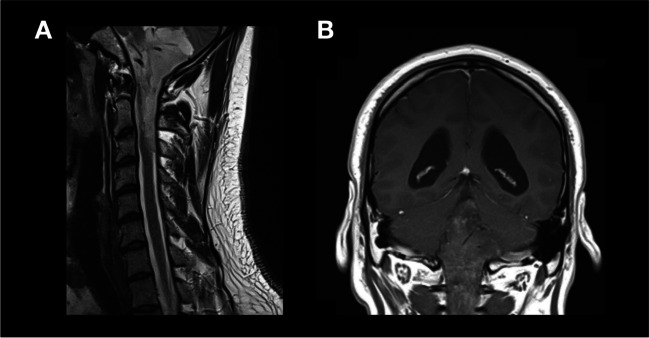
Example of extended craniotomy with C1, and C2 laminotomy in the case of tumor extension inferiorly to the spinal canal

The shape and extent of craniotomy depends on the size and location of the tumor (Fig. 1). In small symmetrical tumors, only the lower part of the occipital bone is exposed. In large tumors, craniotomy starts more superiorly. In the downward direction, the extent of craniotomy depends on the position of the cerebellar tonsils, considering also the possibility of C1 laminectomy or rarely C2 laminotomy in cases of tumor extension inferiorly to the spinal canal (Fig. 2). In cases of asymmetrical tumors reaching the foramen of Luschka or even the CPA, the craniotomy reaches sigmoid sinus to provide access to the cerebellar tonsil both from the midline and from the CPA.

## Description of the technique

**Fig. 3:**
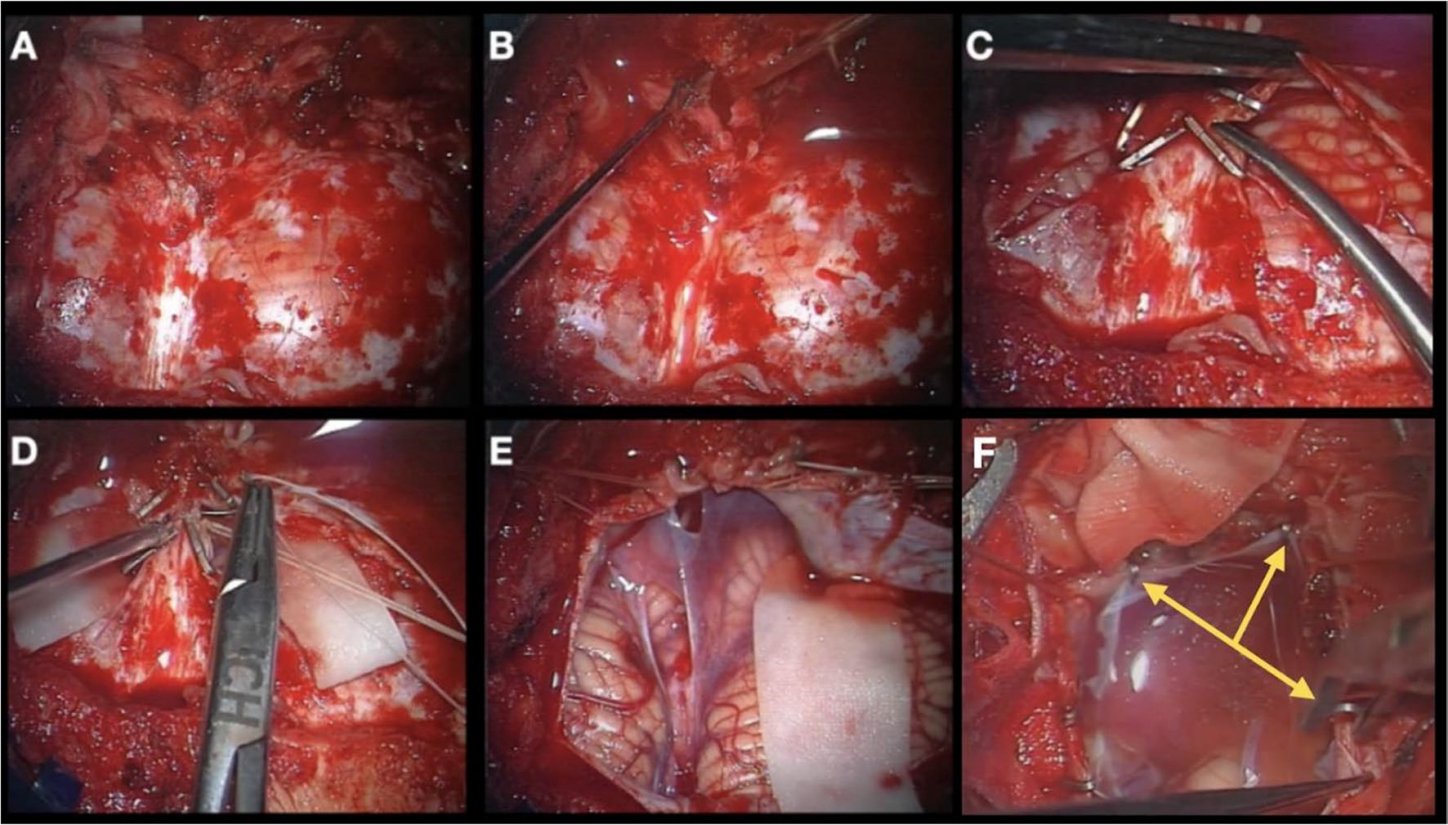
(**A**) Exposure of the dura mater covering both cerebellar hemispheres, (**B**) and its incision. (**C**) Usage of hemoclips for safe sinus occlusion, preventing larger venous bleeding. (**D**) Ligation of the occipital sinus. (**E**, **F**) Elevation of the arachnoid layer and its attachment to the dura superiorly with miniclips (*yellow arrows*)

The dura is opened in “Y” shape. A short linear cut is done below the foramen magnum and CSF is drained. Large hemoclips are used to occlude occipital and marginal sinuses and the cut runs between them. All clips are removed once the dura is reflected. The arachnoid of the cisterna magna is opened superiorly as far as cerebellar vermis and laterally as needed, in the CPA as far as the CP cistern. The arachnoid is elevated and fixed with miniclips to the dura (Fig. 3).

In some cases, the tumor is immediately seen between the tonsils, in some the tonsils need to be dissected in the midline and slightly retracted laterally to expose the lower aspect of the tumor (Video). The distal aspect of the tumor is dissected and elevated from the floor of the IVth ventricle at the calamus area.

The lateral aspects are then addressed, first on the smaller side if the tumor is an asymmetric one. The dissection proceeds from inferior to superior and runs along the tela choroidea which is cut, the vessels (PICA and its branches) are spared and moved laterally. Only the vessels entering the tumor are coagulated and cut. In case the tumor extends into the Luschka foramen, the tonsil is dissected free from both medial and lateral parts and lifted in order to expose the foramen. The dissection then proceeds laterally to the vessels as far as cranial nerves VII–XI. The cerebellar ependyma is usually not well preserved but the tumor can easily be dissected from the cerebellum. If the tumor enters the foramen of Luschka, then the tumor usually forms “a plug” in the foramen. The tumor not extending into the CPA can be resected from the IVth ventricle. If the tumor forms a more voluminous part in the CPA, then this part is approached from lateral, the tonsil is dissected even at its lateral part and the CPA part of the tumor is addressed from outside.

**Fig. 4:**
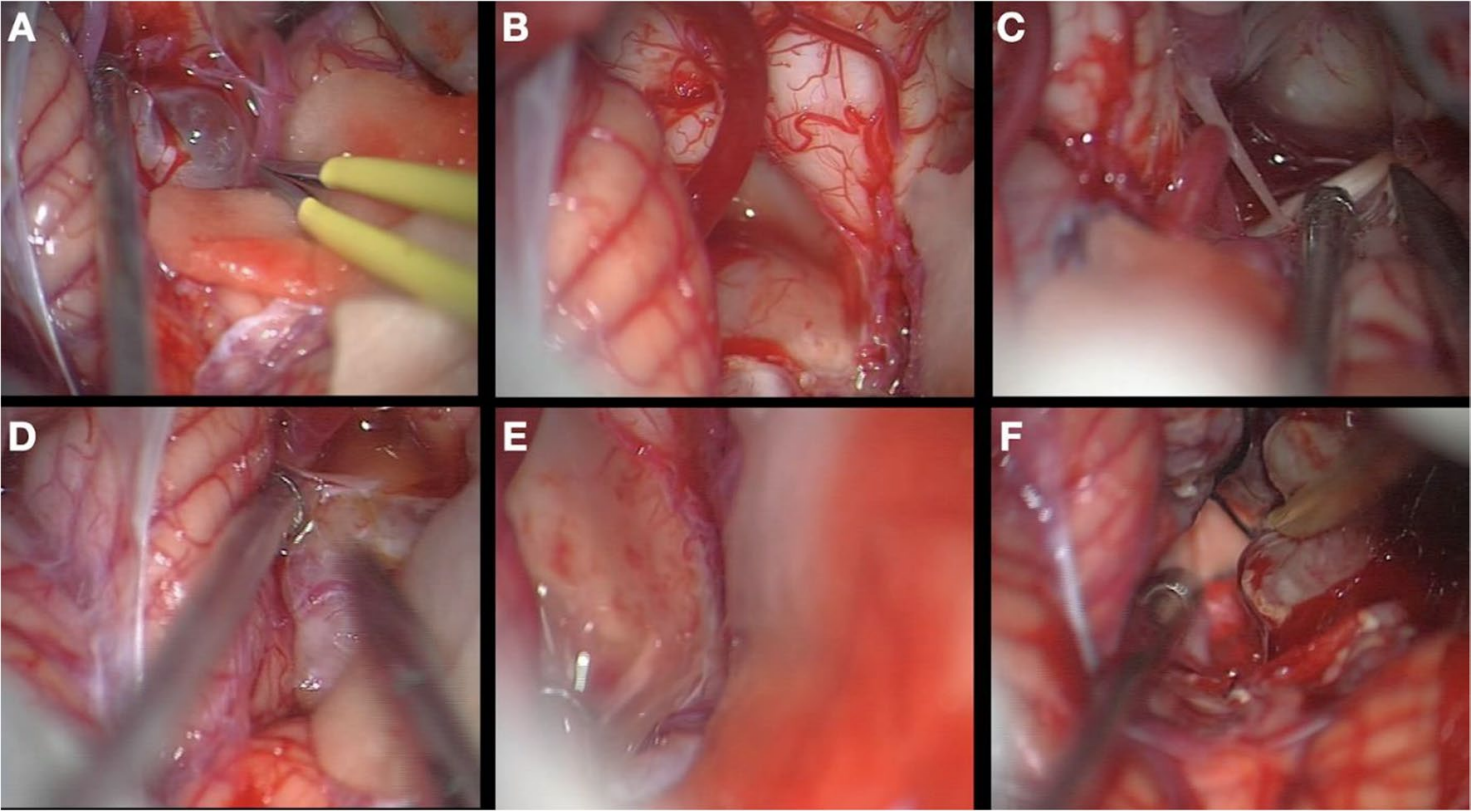
(**A**) Exposure of the cerebellar tonsil along with its movement in order to expose the inferior aspect of the tumor. (**B**) Identification of the rhomboid fossa with resection of the lower part of the tumor. (**C**) Exposure of the CP angle. (**D**) Dissection of the lateral aspects of the tumor (both right and left from the surgical corridor). (**E**) Rhomboid fossa from the lateral view. (**F**) Upper aspect of the tumor identified

**Fig. 5:**
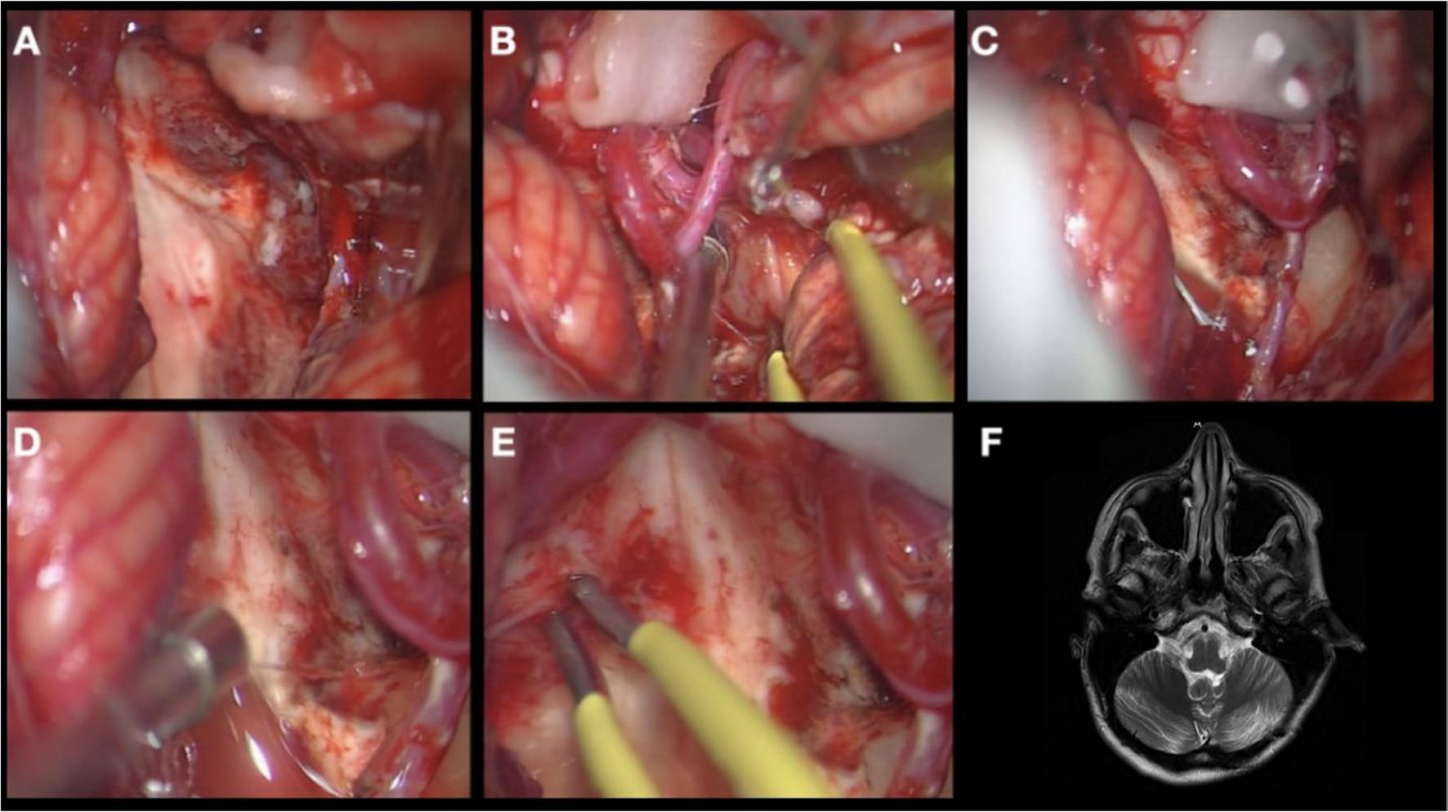
(**A**) The surgical view after the resection of the main bulk of the tumour (see Fig. 3F). (**B**) Tumour remnant in the Luschka foramen. (**C**) Resection of the remnant in the Luschka foramen. (**D**) Inspection of the Luschka foramen, and (**E**) the last film on the lateral proportion of the surgical field resected. (**F**) Postoperative MRI

Once this is done, the superior aspect of the tumor is dissected down to the floor of the ventricle (Fig. 4). The tumor is gradually decompressed by CUSA and once it is dissected free on all sides, it is resected along the margins slowly converging on the part which enters the ependyma on the floor (Fig. 5). Invariably, it is one side of the floor in the lower half of the ventricle, caudal to striae. We have never seen the tumor arising from the floor above the level of the foramen of Luschka. Majority of our tumors had its origin on the right side.

The floor now is mapped, the nuclei of the cranial nerves IX–XII localised and only then the remnant is dissected from the floor. The tumor almost always grows from the ependymal into the ventricle cavity, infrequently into the brainstem (Video). The resection of subependymal lobules is the same as in spinal cord ependymomas (Video). Some bleeding usually occurs. The bipolar is never used to control the bleeding – gentle tamponade, small pieces of SURGICEL® and flushing with saline are used to control the bleeding. All patients underwent routine extubation in the OR unless complications about achieving adequate hemostasis or potential issues with the lower cranial nerves during surgery occurred. In such instances, patients remained intubated overnight, and a trial extubation was performed the next day in the intensive care unit. Following surgery, all patients were moved to the intensive care unit for thorough neuromonitoring.

## Outcomes

The majority of surgeries were gross total (79.2%). Any relapsing disease is the growth of the remnant, which we indicated for radiosurgical treatment in 3 cases of subtotal resection. Overall mortality was 4.2% and morbidity was 8.3%. The mean follow-up was 7.2 ± 6.8 years. In neither of the radically resected tumors did we encounter recurrent disease.

## Limitations

The neuromonitoring and mapping of the rhomboid fossa are mandatory, requiring longer preoperative preparation in the OR. Too close proximity of IX–XIth nuclei and decreased potentials may prevent radical resection.

## How to avoid complications

Meticulous work with arachnoid layers in the CPA prevents the injury of cranial nerves. All perforating arteries from PICA must be identified and saved. To prevent damage to rhomboid fossa, any coagulation should be avoided. Precise mapping of the floor of the IVth ventricle helps to avoid nuclei damage.

## Specific information for the patient about surgery and potential risks

The patient should be familiarized about the risk of neurological deficit or shunt-dependency after surgery.

## Videos legends

Two illustrative videos are included in this manuscript with commented presentation of two case reports. Treatment algorithm and both surgical and clinical results are introduced.

## Data Availability

The whole dataset is available from the corresponding author upon reasonable request.
